# Inhibition of Nur77 expression and translocation by compound B6 reduces ER stress and alleviates cigarette smoke-induced inflammation and injury in bronchial epithelial cells

**DOI:** 10.3389/fphar.2023.1200110

**Published:** 2023-06-19

**Authors:** Chenli Chang, Fengming He, Mingtao Ao, Jun Chen, Tao Yu, Weiyu Li, Baicun Li, Meijuan Fang, Ting Yang

**Affiliations:** ^1^ China-Japan Friendship Hospital, Center of Respiratory Medicine, National Center for Respiratory Medicine, National Clinical Research Center for Respiratory Diseases, Institute of Respiratory Medicine, Chinese Academy of Medical Sciences, Beijing, China; ^2^ School of Pharmaceutical Sciences, Xiamen University, Xiamen, China; ^3^ College of Pharmacy, Hubei University of Science and Technology, Xianning, China

**Keywords:** Nur77, airway epithelial cells, inflammation, apoptosis, small molecule derivative of flavonoid

## Abstract

Chronic obstructive pulmonary disease (COPD) is a leading cause of death worldwide with inflammation and injury in airway epithelial cells. However, few treatment options effectively reduce severity. We previously found that Nur77 is involved in lipopolysaccharide-induced inflammation and injury of lung tissue. Here, we established an *in vitro* model of COPD-related inflammation and injury in 16-HBE cells induced by cigarette smoke extract (CSE). In these cells, Nur77 expression and localization to the endoplasmic reticulum (ER) increased following CSE treatment, as did ER stress marker (BIP, ATF4, CHOP) expression, inflammatory cytokine expression, and apoptosis. The flavonoid derivative, named B6, which was shown to be a modulator of Nur77 in previous screen, molecular dynamics simulation revealed that B6 binds strongly to Nur77 through hydrogen bonding and hydrophobic interactions. Treating CSE-stimulated 16-HBE cells with B6 resulted in a reduction of both inflammatory cytokine expression and secretion, as well as attenuated apoptosis. Furthermore, B6 treatment resulted in a decrease in Nur77 expression and translocation to the ER, which was accompanied by a concentration-dependent reduction in the expression of ER stress markers. Meanwhile, B6 played a similar role in CSE-treated BEAS-2B cells. These combined effects suggest that B6 could inhibit inflammation and apoptosis in airway epithelial cells after cigarette smoke stimulation, and support its further development as a candidate intervention for treating COPD-related airway inflammation.

## Introduction

Chronic obstructive pulmonary disease (COPD), a common chronic respiratory disease, was the third leading cause of death worldwide in 2019, caused by a complex set of environmental factors, primarily including inhalation of particulate matter, cigarette smoke, and air pollutants, along with genetic, developmental, and social factors (Christenson et al., 2022). According to large-scale epidemiological studies such as BOLD and others, the estimated global prevalence of COPD is 10.3% (Adeloye et al., 2015; Adeloye et al., 2022), and its prevalence increases yearly (Venkatesan, 2023). COPD is a heterogeneous disease, characterized by persistent respiratory symptoms and airflow restriction due to abnormalities in the distal airway (Barnes et al., 2015). Its pathological manifestations include varying degrees of chronic bronchitis and damage to the pulmonary parenchyma (Calverley and Walker, 2003).

Cigarette smoke (CS) is known to contain more than 7,000 harmful substances (Soleimani et al., 2022), which can lead to oxidative stress, squamous metaplasia, mucus hypersecretion, ciliary shedding in the airway epithelium, cytokine secretion and subsequent recruitment of immune cells, which cumulatively result in limiting airflow (Yoshida et al., 2019; Dang et al., 2020; Zhou et al., 2020). At the cellular level, exposure to CS compromises the integrity or leads to loss of tight junctions between epithelial cells, resulting in the development of emphysema and subsequent pathogenesis of COPD (Tatsuta et al., 2019). However, the mechanism through which CS exposure leads to COPD development remains unclear.

Nur77, also known as NR4A1, is reportedly involved in asthma and acute lung injury through the activation of inflammatory response *via* regulation of NF-κB signaling (Kurakula et al., 2015; Jiang et al., 2016). Similarly, Nur77 ^−/−^ mice were found to exhibit increased sensitivity to bleomycin and higher susceptibility to pulmonary fibrosis (Palumbo-Zerr et al., 2015). Although the absence of endogenous ligands has led to its annotation as an orphan receptor (Safe et al., 2021), Nur77 is nevertheless widely expressed in different tissues and known to participate in a variety of processes as a transcriptional regulator in the nucleus or interaction partner modulating the function of other proteins (Chen et al., 2019; Niu et al., 2021). In particular, Nur77 has been shown to play roles in inflammatory response, cellular proliferation and differentiation, apoptosis, and autophagy (Koenis et al., 2018; Peng et al., 2021; Ye et al., 2021). Nur77 was also found to contribute to pathological inflammatory responses in diseases such as atherosclerosis, obesity, diabetes, arthritis, inflammatory bowel disease, acute liver inflammation, neuroinflammation, tumor inflammation, and respiratory diseases (Hanna et al., 2012; Wu et al., 2016; Li et al., 2020; Li et al., 2022; Ahuja et al., 2023). These studies collectively highlight the role of Nur77 in disease development and suggest that Nur77 may be an effective target for developing anti-inflammatory drugs. However, the function and mechanism of Nur77 in COPD have been largely overlooked.

Approximately one-third of the proteins in eukaryotic cells are secreted or membrane proteins that depend on the endoplasmic reticulum (ER) for biosynthesis, folding, and post-translational modification (Borges and Lake, 2014). In general, cells maintain a dynamic balance between protein biosynthesis and folding, a process known as ER homeostasis (Schinzel et al., 2019). Endoplasmic reticulum stress (ER stress) is induced by the aberrant accumulation of unfolded proteins in the ER due to the disruption of ER homeostasis by pathological factors such as oxidative stress, nutrient deprivation, ischemia, hypoxia, glucose deprivation, viral infection, or loss of calcium homeostasis. The development of ER stress involves the IRE1, PERK (PEK), and ATF6 pathways, and can result in programmed cell death or injury via apoptosis, autophagy, or ferroptosis (Wang et al., 2021; Zhang et al., 2021). In addition, ER stress can lead to cellular inflammation mediated by the MAPK, NF-κB, and other signaling pathways, which has been shown to contribute to the pathogenesis of several diseases (Zhang et al., 2018; Chen et al., 2022).

In previous work, we found that the flavonoid derivative, B6, could inhibit the development of lipopolysaccharide (LPS)-induced acute lung injury by targeting Nur77 (Ao et al., 2022). However, the effects of B6 on the subcellular localization of Nur77 and downstream ER stress in airway epithelial cells characteristic of COPD-related airway inflammation have not been explored. In this study, we found that Nur77 expression and translocation to the ER is increased in airway epithelial cells following exposure to cigarette smoke extract *in vitro*. These aberrant patterns of expression and localization lead to downstream induction of ER stress, which consequently promotes inflammation and apoptosis in airway epithelial cells. However, treatment with B6 results in decreased Nur77 expression and translocation to the ER, subsequently alleviating inflammation and reducing apoptosis in bronchial epithelial cells exposed to cigarette smoke extract. Molecular dynamics (MD) simulations suggest that B6 directly interacts with Nur77 and support its further exploration for possible application in the treatment of CSE-related inflammation.

## Methods

### Molecular docking

The crystal structure of the Nur77-3NB complex utilized in this study was sourced from the PDB database and had a resolution of 2.18 Å (PDB ID: 4WHG). The protein structure was prepared using the default parameters of the Protein Preparation Wizard panel of the Schrödinger Suite (version 2021-2). The preparation steps involved adding hydrogen atoms, deleting water molecules, adding charges, removing crystal solvents, completing missing residue side chains and loops utilizing the Prime module (Jacobson et al., 2002; Jacobson et al., 2004), optimizing hydrogen bond networks, and performing restrained energy minimization of the protein structure utilizing the OPLS4 force field (Lu et al., 2021). The small molecule ligand was prepared by subjecting it to the LigPrep (LigPrep, Schrödinger, LLC, New York, NY, 2021) tool of Schrödinger with default parameters, thereby converting its 2D structure to a 3D structure. Thereafter, molecular docking was conducted using the Induced Fit Docking panel (Induced Fit Docking, Schrödinger, LLC, New York, NY, 2021) of Schrödinger. The native ligand 3NB served as the docking box center, and default parameters were applied under the standard protocol. The top-ranking docking conformation was selected based on the binding mode and docking score for subsequent MD simulations.

### Binding pose metadynamics simulation

Before conducting the all-atom MD simulation, we performed a 10 × 10 ns binding pose metadynamics (BPMD) stimulation to evaluate the binding stability of B6 and Nur77. We utilized the Binding Pose Metadynamics panel of Schrödinger with default parameters, which involved selecting the Nur77-B6 complex as the structure type and setting the number of trials per pose to 10. The time series plot of the collective variable root-mean-square deviation (CV RMSD) was then obtained to analyze the reliability of the selected docking pose.

### Molecular dynamics simulation

The B6/Nur77 complex was subjected to an all-atom MD simulation with periodic boundary conditions utilizing the OPLS4 force field (Lu et al., 2021) within the Desmond software (Desmond, Schrödinger, LLC, New York, NY, 2021) (Bowers et al., 2006; Desmond, 2021). The simulation system was constructed using the System Builder module of Desmond and solvated within a periodic cubic box. The distance between the box boundary and the complex was maintained at a minimum of 10 Å. The simulation system employed the predefined TIP3P water model and was neutralized by the inclusion of K^+^ and Cl^−^ ions. Sufficient K^+^ and Cl^−^ ions were added to achieve a KCl salt concentration of 0.15 M in the simulation system. Prior to the final simulation, a sequence of restrained minimization and MD simulations were executed to equilibrate the system (He et al., 2023). The final MD production was executed in the NPT (constant number of atoms *N*, pressure *P*, and temperature *T*) ensemble. The Nose-Hoover chain thermostat and Martyna-Tobias-Klein barostat were utilized to maintain a constant temperature of 310 K and a pressure of 1.01325 bar, respectively. The trajectory was recorded every 100 ps, and the simulation lasted for 100 ns. The Simulation Interaction Diagram tool was employed to analyze the simulation trajectory, including the RMSD of protein backbone atoms and small molecule ligand heavy atoms, and the analysis of ligand-protein interactions. The binding free energy between B6 and Nur77 was computed the obtained trajectory using the thermal_mmgbsa.py script within Schrödinger. PyMOL (The PyMOL Molecular Graphics System, Version 2.3 Schrödinger, LLC) was used to render overlay plots of the MD trajectory.

### Surface plasmon resonance (SPR)

Following the same procedure in our previous study (Ao et al., 2022). A BIAcore T200 instrument (GE Healthcare) was used in the SPR study. The binding kinetics of Nur77-LBD and B6 were analyzed using the BIAcore T200 (GE Healthcare) at 25°C. The screening concentration was from 0.28 to 10 μM. The negative control was phosphate-buffered saline (PBS). Nur77-LBD proteins were diluted to 0.4 mg/mL in NaOAc (pH 4.5) and immobilized using amine coupling at 6,000 receptor units (RU) on a CM5 sensor chip (GE Healthcare). B6 was injected into the flow wells in running buffer (PBS, 0.1% DMSO) at a flow rate of 30 mL/min for 120 s of association, then dissociated for 420 s. The data was analyzed using the BIAcore T200 Evaluation Software 2.0. The dissociation constant (*K*_D_) was calculated using kinetic data from gradient concentrations fitted to a 1:1 interaction model.

### Fluorescence quenching assay

Different concentrations of B6 were added to Nur77-LBD proteins, and fluorescence quenching was monitored at 25°C with 10 nm slit widths for excitation and 10 nm slit widths for emission. A wavelength of 284 nm was used for excitation, and a wavelength of 450 nm was used for emission. A binding affinity was estimated by measuring fluorescence intensities at 332 nm as quencher concentration increased, and in accordance with the standard formula, the values of *K*_D_ were calculated.

### CSE preparation

CSE was prepared as previously described (Xu et al., 2018). Briefly, a vacuum suction device was used to draw smoke from Marlborough cigarettes (Philip Morris, United States) into glass tubes containing 10 mL of room-temperature Dulbecco’s Modified Eagle Medium (DMEM) (Gibco, United States) at a constant speed. Each cigarette was continuously aspirated for 5 min. The resulting CSE solution was added to a 96-well Costar plate (200 mL of solution per well) and the absorbance was measured at 320 nm with a microplate reader (Spark^®^, Tecan, Mannedorf, Switzerland). The optical density of the CSE was adjusted to 1.0 in DMEM, and the resultant CSE solution was considered as 100% CSE. The diluted solution was then sterilized with a 0.22 μm pore filter (Millipore, United States). The sterilized solution was further diluted in serum-free medium to the concentrations required for the experiments described below. Dilutions occurred within 1 h of sterilization.

### Cell culture and treatment

The 16-HBE and BEAS-2B cells were cultured in DMEM supplemented with 10% fetal bovine serum (Procell Technology), 2 mM l-glutamine (Gibco), 100 U/mL penicillin (Gibco), and 100 μg/mL streptomycin (Gibco) at 37°C in ambient air supplemented with 5% CO_2_. The cells were incubated with 2% CSE for 24 h to detect the effects of CSE on Nur77 concentrations, ER stress markers, and inflammatory cytokine levels. Other cells were stimulated with 5% CSE, then apoptosis was measured by CCK-8 assay and flow cytometry. To investigate the effects of B6, several concentrations (1, 2, 3, and 5 μM) were added to separate samples at 1 h after CSE stimulation. The assays described above were then conducted after 24 h of co-treatment.

### Quantitative reverse transcription-PCR (RT-qPCR)

16-HBE and BEAS-2B cells were inoculated into 24-well plates and co-treated with CSE and B6. The supernatant was removed and cells were washed three times with PBS. TRIzol reagent (Takara Biomedical Technology, Beijing) was added to each well (1 mL each) and the plates were incubated at room temperature for 5 min. Chloroform (0.2 mL per well) was added and the plates were shaken vigorously. After incubation for 15 min on ice, cells were centrifuged at 12,000 rpm to extract RNA from the aqueous phase. The upper liquid containing RNA was isolated and mixed with an equal volume of isopropyl alcohol, then incubated on ice for 10 min. After centrifugation, the supernatant was discarded. The resulting RNA was washed with 1 mL 75% anhydrous ethanol and resuspended in 20 mL RNase-free water. RNA concentrations were measured with a NanoDrop One (Thermo Fisher Scientific, United States), and RNA quality was assessed using the A260/280 ratio, which was between 1.8 and 2.0 for each sample. RNA samples were stored at -80°C or used as cDNA template using a reverse transcription kit following the manufacturer’s instructions (PrimeScript™ RT Master Mix, Takara Biomedical Technology, Beijing). RT-qPCR was performed with an real time PCR kit as instructed by the manufacturer to detect mRNA levels of *IL-6*, *IL-8*, *IL-1*β, and *TNFα* (TB Green^®^ Premix Ex Taq™, Takara Biomedical Technology, Beijing).

### Western blot

Cell lysates were prepared in radioimmunoprecipitation assay (RIPA) buffer containing 50 mM Tris (pH 8.0), 150 mM NaCl, 1% Triton X-100, 1 mM EDTA, 0.5% sodium deoxycholate, and 0.1% sodium dodecyl sulfate (SDS) (Solarbio, Beijing). The supernatant and precipitation fractions were obtained via centrifugation. The proteins were then separated with 10% SDS-polyacrylamide gel electrophoresis (PAGE) (Shanghai Epizyme Biomedical Technology) and transferred to a PVDF membrane (Merck KGaA, Darmstadt, Germany). The following primary antibodies were used: anti-Nur77 (CST, 3960, 1:1000), anti-CHOP (CST, 2895, 1:1000), anti-BIP (CST, 3177, 1:1000), anti-ATF4 (Proteintech, 10835-1-AP, 1:1000) and anti-cleaved caspase3 (Immunoway, YC0006, 1:1000). The membrane was incubated with primary antibodies overnight at 4°C, then with secondary antibodies for 1 h at room temperature. Protein bands were visualized with an enhanced chemiluminescence kit on a ChemiDoc Chemiluminescent Gel Imaging System (Bio-Rad, United States). Individual protein band intensities were quantified with ImageJ software (NIH, United States).

### Enzyme-linked immunosorbent assay (ELISA)

The supernatant was collected from cultured 16-HBE cells after 24 h CSE stimulation. Levels of secreted IL-6 and IL-8 were measured in the collected culture medium using the Human Interleukin 6 (IL-6) ELISA Kit and the Human Interleukin 8 (IL-8) ELISA Kit (Invitrogen, Thermo Fisher Scientific, United States) following the manufacturer’s instructions. After reaction termination, sample absorbances were detected at 450 nm using a microplate reader (Spark^®^, Tecan, Mannedorf, Switzerland). The standard curve was generated using the concentrations of the standards and the associated OD values. Finally, IL-6 and IL-8 concentrations in the samples were calculated using the standard curve.

### Immunofluorescence

Equal numbers of 16-HBE cells were seeded on 14-mm glass coverslips pretreated with TC (Tissue culturetreated) (NEST, Jiangsu). After culturing for 24 h to reach 90% confluency, cells were treated with vehicle or test compounds for 24 h. The supernatant was discarded, then cells were washed with PBS. Cells were fixed in 4% paraformaldehyde for 15 min, which was followed by three consecutive washes with PBS. Cells were permeabilized with 0.1% Triton X-100 in PBS for 20 min, washed three times with PBS, and incubated with 10% goat serum for 1 h. These steps were conducted at room temperature. Cells were then incubated with 10% goat serum containing a 1:200 dilution of Nur77 primary antibody at 4°C overnight, then washed three times with PBS for 5 min each. Cells were incubated with the red-labeled antibody IFKine™ Red Donkey Anti-Rabbit IgG (1:200, Abbkine Scientific Co., United States) for 2 h at room temperature. Cells were then incubated with a 1:200 dilution of the green-labeled ER-tracker (Beyotime, C1042) at 37°C for 30 min so the ER membranes could be visualized. After washing 3 times with PBS, cells were stained with DAPI (C1005) (1:1,000) for 5 min so the cell nuclei could be visualized. Glass coverslips were removed from the dishes, then cells were inverted on glass slides and mounted with antifading mounting medium (S2100, Solarbio). Images were captured with an image microscope (Nikon, Japan).

### Flow cytometry

Six-well plates (Corning, New York, United States) were seeded with 16-HBE cells at a concentration of 2 × 10^5^ cells per well. At 24 h after seeding, cells were treated with vehicle or test compounds and incubated for 24 h. Cells were then digested with trypsin to prepare single-cell suspensions. The digests were centrifuged and resuspended in antibody-binding buffer, then the cells were counted. Annexin V-FITC (4A biotech, Suzhou) (5 mL per sample) was added to each sample of 1 × 10^5^ resuspended cells and mixed gently. Samples were incubated for 10 min in the dark at room temperature. After centrifugation, the supernatant was discarded, and cells were resuspended in 100 mL of antibody binding buffer. Propidium iodide staining solution (4A biotech, Suzhou) (10 mL) was added to each sample and gently mixed well, then staining was immediately halted by adding PBS. Flow cytometry was performed immediately (Beckman Coulter, United States).

### Cell viability assay

Cell viability assays were conducted for 16-HBE and BEAS-2B cells using CCK8 bioassay. Briefly, 5 × 10^3^ cells per well were seeded into 96-well plates to adhere overnight. Vehicle or test compounds were added and incubated for 24 h, after which 100 mL of complete medium with 10 mL CCK solution was added to each well and incubated for 1–4 h according to the appropriate OD value. Finally, absorption values were measured at 450 nm with a spectrophotometer (Spark^®^, Tecan, Mannedorf, Switzerland). Cell viability rates were calculated as follows:

$$viability\;rate\;\left(\%\right)=OD\left(experimental\;group\;–\;blank\;well\;/\;control\;group\;–\;blank\;well\right)\times 100\%$$

### Statistical analysis

For each experiment, the mean values and standard deviation were calculated from at least three independent replicates. Differences between groups were analyzed with one-way analysis of variance (ANOVA). Differences were considered statistically significant at *p* < 0.05. All statistical analyses were performed in GraphPad Prism 9. *p < 0.05, **p < 0.01, ***p < 0.001.

## Results

### Nur77 is involved in cigarette smoke-induced injury and inflammation in epithelial cells

**FIGURE 1:**
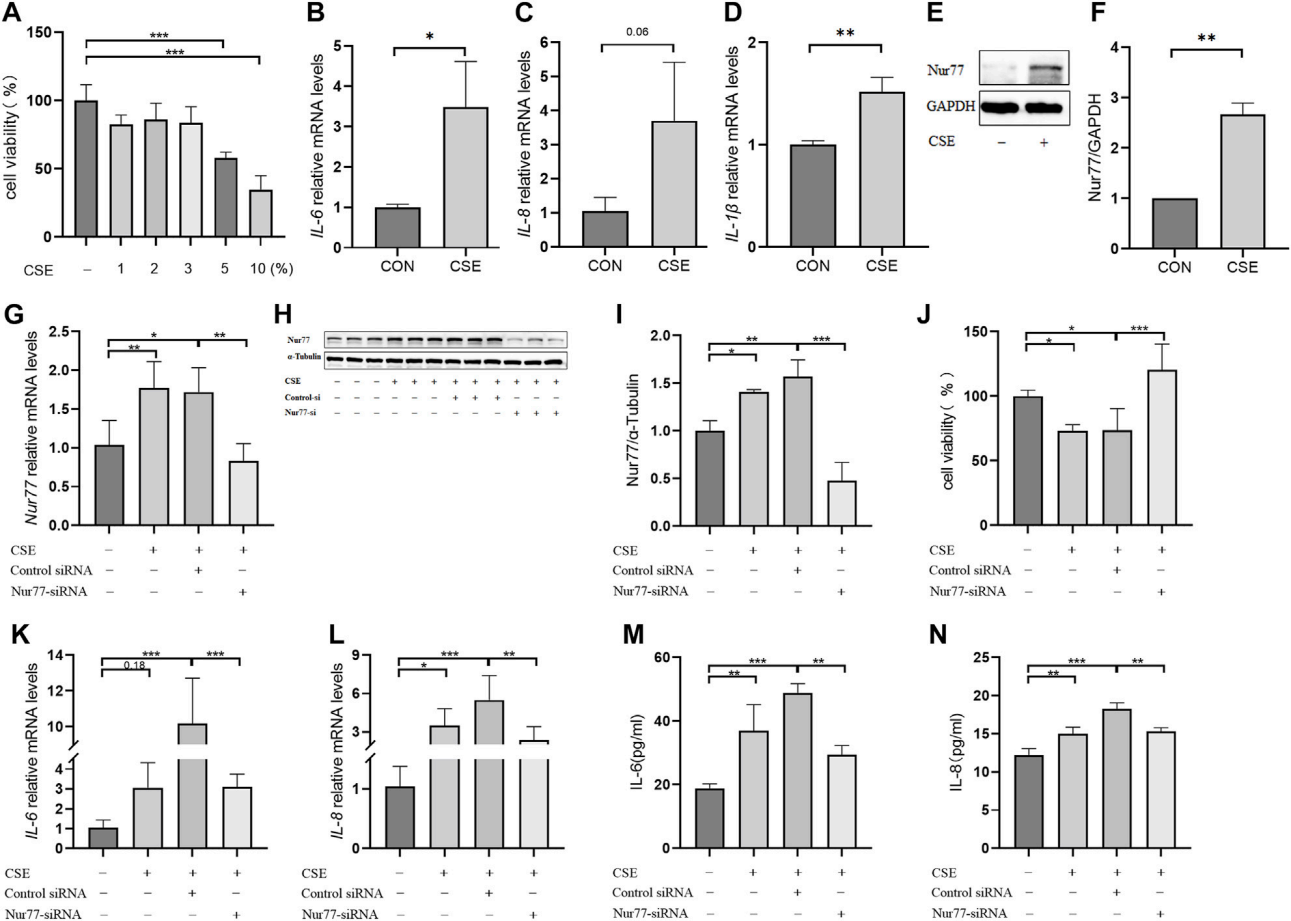
Nur77 plays an important role in cigarette smoke-induced epithelial cell inflammation and injury **(A)** Evaluation of 16-HBE cell viability after stimulation with several concentrations of cigarette smoke extract (CSE). Cell viability was measured with a Cell Counting Kit-8 (CCK8) bioassay. **(B–D)** mRNA levels of *IL-6* **(B)**, *IL-8* **(C)**, and *IL-1*β **(D)** after 2% CSE stimulation as determined with reverse transcription (RT)-qPCR. **(E, F)** Nur77 protein levels after 2% CSE stimulation as detected with western blot (WB). **(G–I)** Transfection efficiency of Nur77 short interfering (si)RNA in 16-HBE cells as determined with RT-PCR **(G)** and WB **(H, I)**. **(J)** Effects of 5% CSE on 16-HBE cell viability after transfection with Nur77 siRNA as determined with a CCK8 bioassay. **(K, L)** mRNA levels of *IL-6* **(K)** and *IL-8* **(L)** in 16-HBE cells transfected with Nur77 siRNA after 2% CSE stimulation as determined with RT-PCR. **(M, N)** Levels of IL-6 **(M)** and IL-8 **(N)** secreted by 16-HBE cells transfected with Nur77 siRNA after stimulation with 2% CSE as determined with enzyme-linked immunosorbent assay (ELISA). Data are presented as the least squares mean ± standard deviation. *p < 0.05, **p < 0.01, ***p < 0.001 (one-way analysis of variance).

In order to better understand the role of Nur77 in cigarette smoke-related injury in human bronchial epithelial cells, we measured the viability of cultured 16-HBE cells treated with different concentrations (1%, 2%, 3%, 5%, or 10%) of cigarette smoke extract (CSE) *in vitro* by CCK8 assays. While viability was significantly decreased in the 5% and 10% treatment groups, cells treated with 1%, 2%, or 3% CSE showed a non-significant decreasing trend in the proportion of viable cells compared to controls (Figure 1A). In light of the above data, we selected 2% CSE to examine the transcriptional effects of CSE without inducing cell death in a significant proportion of our cultures (Maremanda et al., 2021). Following 24 h incubation with 2% CSE, we used RT-qPCR to measure mRNA levels of the inflammatory factors *IL-6* (Figure 1B), *IL-8* (Figure 1C) and *IL-1*β (Figure 1D), all of which were increased after CSE stimulation compared with that in untreated controls. Previous study of Nur77 in acute lung injury confirmed that Nur77 plays an important role in airway inflammation (Ao et al., 2022), we examined Nur77 expression in these 16-HBE cells after CSE stimulation. Western blotting showed that Nur77 protein accumulated to significantly higher levels after CSE treatment than that in untreated control cells (Figure 1E, quantified in Figure 1F).

To investigate the potential role of *Nur77* in cigarette smoke-induced inflammation in epithelial cells, we synthesized siRNAs targeting Nur77 and non-targeted RNA scramble controls, and transfected 16-HBE cells with 50 nM siNur77 or RNA scramble controls at a cell confluency of 50%. The medium was removed at 6 hours post-transfection and cells were stimulated with 2% CSE. RT-qPCR assays showed that the CSE-induced increase in *Nur77* mRNA levels was abolished in the si-Nur77 transfected cells, with transcript levels lower than that in untreated control cells (Figure 1G), which was verified by Western blot detection of Nur77 protein (Figure 1H, quantified in 1I). CCK8 assays further indicated that Nur77 knockdown (KD) could attenuate the CSE-induced reduction in cell viability (Figure 1J), While RT-qPCR and ELISA assays indicated that both transcriptional expression and secretion of inflammatory factors were inhibited in Nur77 KD cells (Figures 1K–N). These results suggested that Nur77 participates in cigarette smoke-induced inflammation and injury of bronchial epithelial cells.

### Nur77-activated ER stress leads to apoptosis and inflammatory processes in airway epithelial cells

**FIGURE 2:**
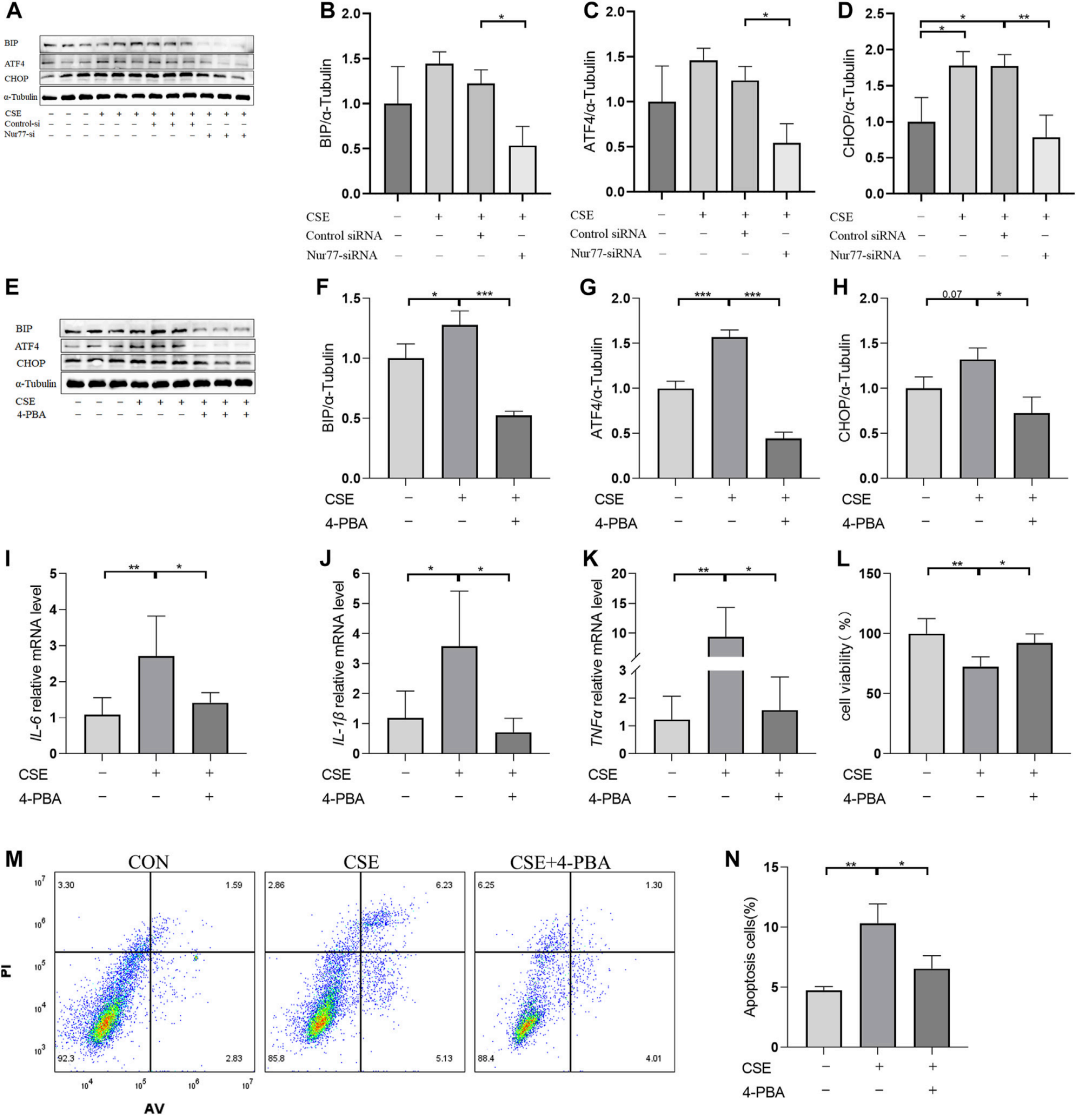
Endoplasmic reticulum stress, activated by Nur77, plays an important role in apoptosis and inflammation of airway epithelial cells **(A–D)** Protein expression levels of BIP (A, quantified in **(B)**, ATF4 (A, quantified in **(C)**, and CHOP (A, quantified in **(D)** in cells transfected with Nur77 short interfering (si)RNA after 2% cigarette smoke extract (CSE) stimulation as detected with western blot. **(E–H)** Protein expression levels of BIP (E, quantified in **(F)**, ATF4 (E, quantified in **(G)**, and CHOP (E, quantified in **(H)** in 16-HBE cells treated with 2% CSE and 4-PBA as detected with western blot. **(I–K)** mRNA expression levels of *IL-6* **(I)**, *IL-1*β **(J)**, and *TNF-*α **(K)** after treatment with 2% CSE and 4-PBA as detected with reverse transcription (RT)-PCR. **(L)** Viability of 16-HBE cells after treatment with 5% CSE and 4-PBA as measured with a CCK8 bioassay. **(M, N)** Apoptosis rates among 16-HBE cells after treatment with 5% CSE and 4-PBA as determined with flow cytometry. Data are presented as the least squares mean ± standard deviation. *p < 0.05, **p < 0.01, ***p < 0.001 (one-way analysis of variance).

Previous studies have confirmed that ER stress plays an important role in inflammation and apoptosis (Choi et al., 2020). Therefore, we investigated whether the upregulation of Nur77 in airway epithelial cells following CSE exposure also contributed to ER stress and further supported the role of ER stress in CSE-induced airway inflammation and injury via a specific inhibitor of ER stress, 4-PBA. Western blot analysis showed that treatment with 2% CSE resulted in significantly higher protein levels of the ER stress marker proteins, BIP (Figure 2A, quantified in Figure 2B), ATF4 (Figure 2A, quantified in Figure 2C)and CHOP (Figure 2A, quantified in Figure 2D), but not in the siNur77 cells, suggesting that Nur77 was also involved in CSE-induced ER stress. Next, we pretreated 16-HBE cells with 4-PBA for 2 h and then stimulated them with CSE to detect the indicators of ER stress. Western blotting indicated that BIP (Figure 2E, quantified in Figure 2F), ATF4 (Figure 2E, quantified in Figure 2G), and CHOP (Figure 2E, quantified in Figure 2H) protein levels were significantly lower in CSE-exposed cells pretreated with 4-PBA compared to that in CSE-stimulated without ER stress inhibitor. In addition, mRNA expression of the inflammatory factors *IL-6* (Figure 2I), *IL-1*β (Figure 2J), and *TNFα* (Figure 2K) was significantly lower in CSE-treated 16-HBE cells pre-treated with 4-PBA compared to their expression under CSE stimulation alone, suggesting that blocking ER stress could alleviate CSE-induced inflammation. To further investigate whether ER stress led to apoptosis in CSE-treated cells, we evaluated the effects of 4-PBA on apoptosis after CSE treatment using CCK8 assays (Figure 2L) and flow cytometry (Figures 2M,N). Both experiments showed that 4-PBA could reduce the proportion of apoptotic 16-HBE cells induced by CSE. These collective results supported the likelihood that cigarette smoke exposure could activate ER stress through Nur77 upregulation, resulting in airway inflammation and apoptosis in bronchial epithelial cells, and thus suggesting a role in the pathogenesis of COPD.

### Hydrogen bonding and hydrophobic interactions mediate the high binding affinity of B6 with the Nur77 ligand binding domain

**FIGURE 3:**
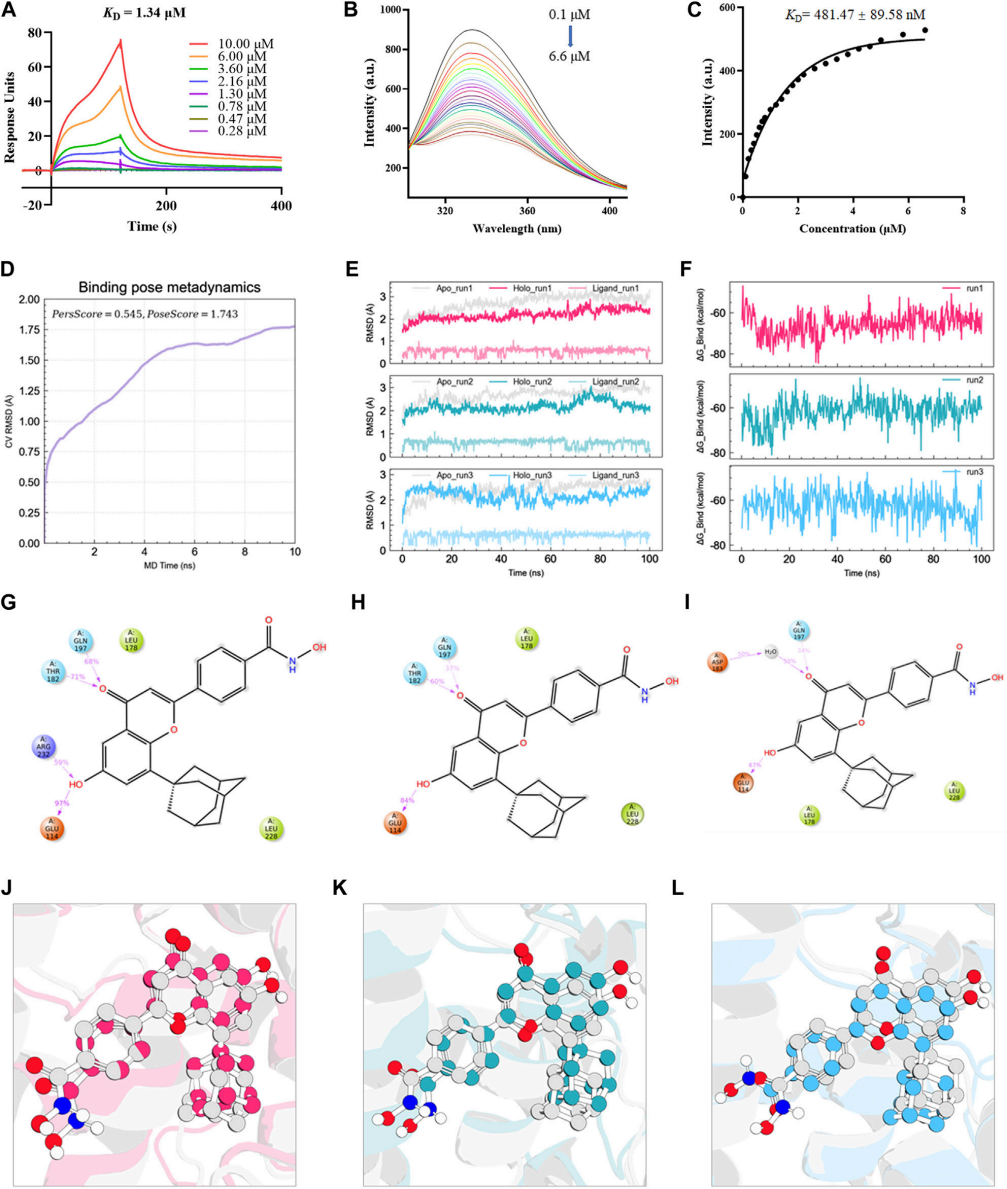
B6 exhibits high binding affinity with the NUR77-LBD through hydrogen bonding and hydrophobic interactions **(A)** An SPR experiment with purified Nur77-LBD showed binding of B6 to Nur77. **(B, C)** Fluorescence titration curve of Nur77-LBD with B6. The inhibitor concentration was increased from 0.1 μM to 6.6 μM at 0.1 μM intervals. **(D)** Binding pose metadynamics simulation. **(E)** Time series analysis of RMSD of protein backbone and ligand heavy atoms (three independent replicates). **(F)** Variation of the binding free energy between B6 and Nur77 over time. **(G–I)** Schematic representation of the interaction between B6 and Nur77 during the 100 ns MD simulation (three independent replicates). The percentage value indicates the ability to form hydrogen bond interactions during the simulation time. **(J–L)**. Superposition of the initial conformation and final conformation of the MD trajectory (three independent replicates). The initial conformation of B6 is depicted as a silver ball-and-sticks, while the last frames of the MD trajectory are represented as magenta (run1), cyan (run2), and sky-blue (run3) ball-and-sticks.

Based on a previous screen of candidate Nur77 modulators (Ao et al., 2022), we next examined the effects of the small molecule flavonoid derivative, B6, on cell inflammation and apoptosis following CSE exposure. To this end, we synthesized B6, characterized its binding affinity to Nur77, and evaluated its potential cytoxicity. To confirm that B6 could physically bind to the Nur77-LBD, we performed SPR experiments *in vitro*. The results showed that B6 could indeed bind the Nur77-LBD in a dose-dependent manner, with a dissociation constant (*K*_D_) value of 1.34 μM, and relatively fast association/dissociation reaction kinetics (Figure 3A). Furthermore, the intrinsic fluorescence intensity of the Nur77-LBD (284 nm excitation/332 nm emission) was significantly reduced in the presence of B6 (Figure 3B) in a dose-dependent manner, with a calculated *K*_D_ value of 481.47 ± 89.58 nM (Figure 3C). These results supported that the flavonoid derivative B6 could directly and efficiently bind to Nur77-LBD *in vitro*.

We utilized molecular docking and MD simulations to investigate the interaction between B6 and Nur77. Initially, the induced-fit docking method was used to dock B6 to the active site of Nur77’s ligand-binding domain (Nur77-LBD), and the top-ranked docking conformation was selected for subsequent MD analysis. To evaluate the stability of ligand-protein binding in aqueous solution during short MD simulations, we conducted BPMD simulation, a variant of metadynamics. It is considered that a PersScore ≥0.6 as an indicator of stable and sustained hydrogen bond interactions between the ligand and the protein in aqueous environment, and a PoseScore ≤2 as an indicator of stable ligand-protein binding in aqueous solution. The BPMD simulation results of B6/Nur77 complex demonstrated stable binding of B6 to the active site of Nur77 in aqueous environment (PoseScore = 1.743) with relatively persistent hydrogen bond interactions with the amino acid residues around the binding site, with a PersScore of 0.545 (Figure 3D). In addition, we performed three independent 100 ns all-atom MD simulations to confirm the stability of the B6/Nur77 complex. Figure 3E shows that the RMSD of protein backbone atoms in the simulation system with B6 bound (Holo) was smaller than that without ligand (Apo), and the RMSD of heavy atoms of B6 was relatively stable, fluctuating around 0.6 Å. The binding free energies between B6 and Nur77-LBD in three independent MD simulation runs were −65.86 ± 5.46, −59.94 ± 5.38, and −60.30 ± 5.99 kcal/mol (average of -62 kcal/mol), indicating a high binding affinity between B6 and Nur77 (Figure 3F).

Through analysis of the simulation trajectories, it was observed that B6 mainly forms hydrogen bond and hydrophobic interactions with key residues in the active site of Nur77. Figures 3G–I demonstrates that B6 can form stable and strong hydrogen bond interactions with the THR182, GUN197, GLU114 and ARG232. Notably, B6 can form hydrogen bond interactions with GLU114 and GUN197 for more than 30% of the simulation time in all three independent simulations, indicating the stability and importance of these hydrogen bonds. Moreover, the phenyl ring and adamantane of B6 can form hydrophobic interactions with LEU178 and LEU228, further stabilizing the binding of the small molecule to the protein. Finally, we overlaid the initial and final conformations of the MD trajectories to visually study the conformational changes of the small molecule during the MD simulations. Figures 3J–L shows that after 100 ns MD simulation, B6 can stably bind to Nur77-LBD and interact strongly with key residues through non-covalent bonds, with an RMSD of heavy atoms of B6 in run1, 2, and 3 systems of 0.8488, 1.1609, and 1.2450 Å, respectively.

### B6 significantly reduces cigarette smoke extract-induced inflammation and apoptosis *in vitro*

**FIGURE 4:**
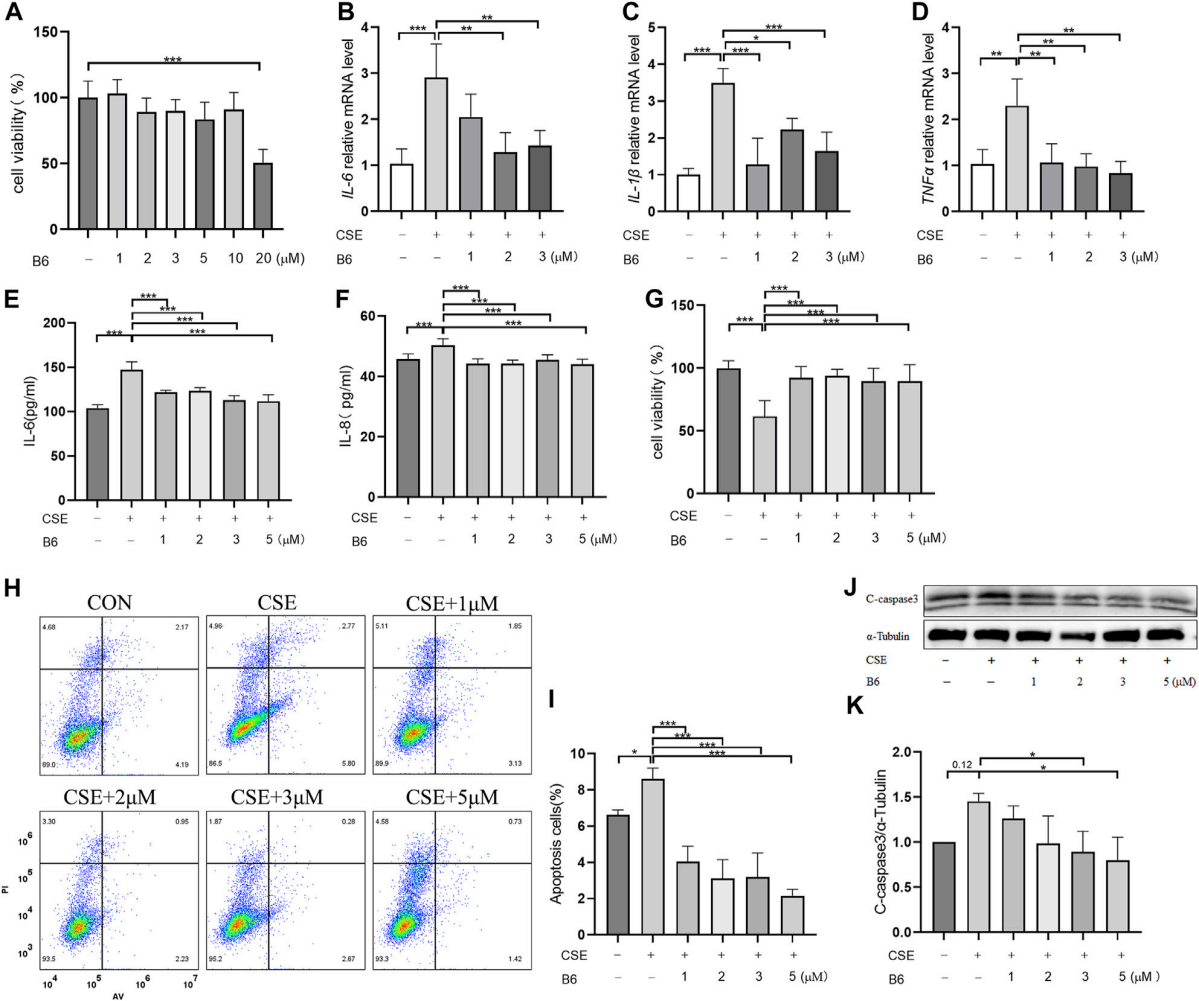
B6 significantly reduces the cellular inflammation and injury caused by cigarette smoke exposure **(A)** Effects of B6 on the viability of 16-HBE cells *in vitro* as determined with a CCK8 bioassay. **(B–D)** rt-PCR was used to detect the mRNA levels of inflammatory cytokines after 2% CSE stimulation with different concentrations of B6 in 16-HBE cells. **(E, F)** ELISA was used to detect the levels of inflammatory cytokines IL-6 **(E)** and IL-8 **(F)** in the medium of 16- HBE cells after 2% CSE stimulation with different concentrations of B6. **(G)** CCK8 was used to determine the viability of 16-HBE cells stimulated by 2% CSE with different concentrations of B6. **(H, I)** Flow cytometry was used to detect the apoptosis percentages of 16-HBE cells after 5% CSE stimulation with different concentrations of B6. **(J, K)** Western Blot was used to detect the protein levels of C-caspase3 [**(J)**, quantified in **(K)**] in 16-HBE cells after the intervention of B6 with different concentrations and CSE. Data are least squares means ± standard errors. *p < 0.05; **p < 0.01,***p < 0.001.

Based on the above interactions between B6 and Nur77, we next examined the effects of various concentrations of B6 on 16-HBE airway epithelial cell viability using CCK8 bioassays. Calculation of IC_50_ values showed that viability was not significantly reduced compared to untreated controls at concentrations of ≤10 mM (Figure 4A; Table 1). To further evaluate whether B6 might be an effective intervention for COPD, we examined the effects of various concentrations of B6, from 1 to 3 μM, in 16-HBE cells at 1 h after CSE stimulation. Relative expression assays using RT-qPCR to quantify *IL-6* (Figure 4B), *IL-1*β (Figure 4C) and *TNF-*α (Figure 4D) transcription showed that treatment with B6 resulted in significantly lower expression following CSE treatment, while ELISA assays indicated that IL-6 (Figure 4E) and IL-8 (Figure 4F) levels were also reduced in cells stimulated with ≥1 μM B6. Moreover, CCK8 assays indicated that the decline in cell viability decline induced by CSE could be rescued by treatment with B6 (Figure 4G), while flow cytometry the proportion of 16-HBE cells with CSE-induced apoptosis was also markedly reduced in cells treated with B6 (Figures 4H,I). In addition, as a typical marker of apoptosis, we used Western blot to detect the protein level of cleaved-caspase3 in 16-HBE cells, as shown in Figures 4J,K, a significant increase in cleaved-caspase3 was observed after CSE stimulation, indicating increased apoptosis. B6 significantly inhibits the expression of cleavage-caspase3, consistent with the flow cytometry results, further supporting the inhibitory effect of B6 on CSE-stimulated apoptosis in 16-HBE cells. These results cumulatively supported that B6 could reduce CSE-induced inflammation and apoptosis.

**TABLE 1:** IC_50_ values of B6 against different epithelial cell lines.

Cell lines	IC_50_ (μmol/L)
16-HBE	19.63 ± 2.46
BEAS-2B	18.05 ± 1.86
MLE-12	10.25 ± 0.98

^1^ IC_50_ values were determined in triplicate (*n* = 6) and expressed as mean ± standard deviation (SD), meaning that the concentration at which B6 inhibited cell viability to 50% was measured using the CCK8 assay.

### B6 inhibits ER stress by affecting Nur77 production and ER localization

**FIGURE 5:**
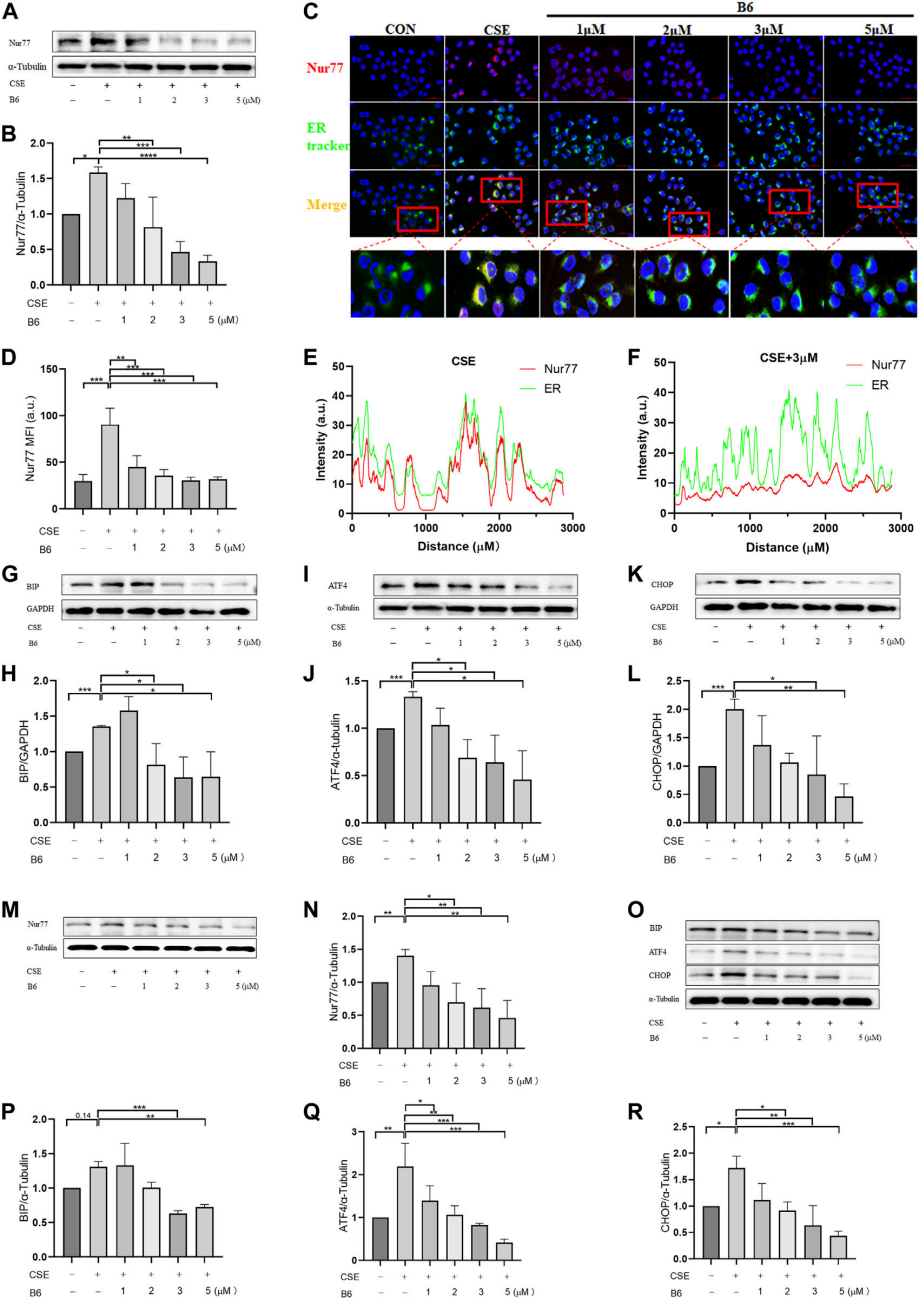
B6 plays a role in the production and subcellular localization of Nur77 under CSE stimulation **(A, B)** Western Blotting was used to detect the protein levels of Nur77 after the intervention of B6 with different concentrations and 2%CSE. **(C)** The co-localization of Nur77 (red) and ER tracker (green) was determined by immunofluorescence; **(D)** Statistical analysis of average fluorescence intensity of Nur77 in different groups; **(E, F)** Statistical analysis of colocalization between Nur77 (red) and ER tracker (green). Western Blotting was used to detect the BIP **(G, H)**, ATF4 **(I, J)** and CHOP **(K, L)** protein level in 16-HBE cells after the stimulation of 2% CSE with different concentrations of B6. **(M, N)** Protein expression levels of Nur77 [**(M)**, quantified in **(N)**] in BEAS-2B cells treated with 2% CSE and different concentrations of B6 as detected with Western blot. **(O–R)** Western blot was used to detect the protein expression of BIP [**(O)**, quantified in **(P)**], ATF4 [**(O)**, quantified in **(Q)**] and CHOP [**(O)**, quantified in **(R)**] in BEAS-2B cells stimulated by 2% CSE and different concentrations of B6. Data are least squares means ± standard errors. *p < 0.05; **p < 0.01,***p < 0.001.

To further investigate the mechanism by which B6 affected cell inflammation and apoptosis, we examined Nur77 expression and localization following CSE stimulation in 16-HBE cells treated or not with B6. Western blot analysis indicated that Nur77 protein levels were significantly lower in CSE-stimulated cells treated with B6 compared to that in cells with CSE stimulation alone (Figure 5A, quantified in Figure 5B). Immunofluorescence staining experiments examining the subcellular localization of Nur77 showed an obvious increase in Nur77 signal and strong co-localization with a probe for ER following CSE treatment (Figure 5C), both of which were partially but significantly reduced in cells treated with B6 (Figures 5D–F). These results indicated that the flavonoid derivative, B6, could inhibit Nur77 expression and function, potentially alleviating inflammation and apoptosis by inhibiting Nur77 translocation to the ER.

We then explored the effects of B6 on ER stress through Western blot analysis of BIP, ATF4 and CHOP protein levels. The results showed that BIP (Figure 5G, quantified in Figure 5H), ATF4 (Figure 5I, quantified in Figure 5J), and CHOP (Figure 5K, quantified in Figure 5L) protein accumulation was lower in cells treated with both CSE and B6 than that in cells stimulated only with CSE. These results suggested that B6 acted as modulator of Nur77 to attenuate ER stress. Further, we detected the time-dependent effects of B6 combined with 2% CSE on the protein level of Nur77 and ER stress indicators at 6, 12, 18, and 24 h time points by Western blot. The results showed that Nur77 (Supplementary Figures S1A, B) increased gradually with time after CSE stimulation, as well as CHOP (Supplementary Figures S1A, C), BIP (Supplementary Figures S1D, E) and ATF4 (Supplementary Figures S1D, F). The protein levels of these indexes were suppressed after the addition of B6 in a time-dependent manner. In addition, cell viability was measured by CCK8 bioassay, and it was found that cell viability decreased significantly at both 6 h (Supplementary Figure S1G) and 12 h (Supplementary Figure S1H) after CSE stimulation, while the inhibitory effect of B6 on apoptosis was significant at 12 h (Supplementary Figure S1H). These results indicate that B6 may have a time-dependent role. To confirm that the effect of B6 on 16-HBE cells was not a special case, we cultured BEAS-2B cells, another common human bronchial epithelial cell line. After CSE and different concentrations of B6 were combined to stimulate BEAS-2B cells, Western Blot was used to detect the effects of CSE and B6 on protein level of Nur77 and ER stress indicators. Consistent with the findings in 16-HBE cells, CSE stimulation significantly increased the protein levels of Nur77 (Figures 5M, N), BIP (Figures 5O, P), ATF4 (Figures 5O, Q) and CHOP (Figures 5O, R), which were significantly inhibited by B6 in a dose-dependent manner. As we found, B6 can inhibit the transcriptional level of Nur77 (Supplementary Figure S2A), inflammatory cytokines (Supplementary Figures S2B–E) in BEAS-2B cells after CSE stimulation. Meanwhile, CCK8 assay showed that B6 can rescue the injury of CSE for BEAS-2B cells (Supplementary Figures S2F). The above results in BEAS-2B cells suggest a universal effect of B6 on airway epithelial cells. Combined with our above results, these experiments indicated that B6 inhibits Nu77 translocation into the ER, suppressing the downstream induction of ER stress, and thereby inhibiting the progression of cigarette smoke-related inflammation and apoptosis in airway epithelial cells.

## Discussion

In the present study, we established an *in vitro* cell model of CSE-associated inflammation and injury in the airway by exposing bronchial epithelial cells to CSE. We then investigate the role of Nur77 in the process of CSE-induced cell injury and inflammatory response. We found that CSE exposure increases the overall expression of Nur77 and as well as its translocation to the ER, subsequently activating endoplasmic reticulum stress, which thus contributes to the inflammation and apoptosis in airway epithelium. A screen of small molecules in previous work identified flavonoid derivative B6, as a potential modulator of inflammation. We confirmed this effect and further found through molecular dynamics simulations that B6 can bind with Nur77. Finally, we found that treatment with B6 resulted in lower Nur77 levels, less translocation to the ER, and reduced ER stress in bronchial epithelial cells, supporting that B6 could reduce CSE-related airway inflammation and injury.

As a nuclear receptor, Nur77 has been shown to participate in the regulation of a variety of inflammatory diseases, and is differentially expressed in organs and tissues with chronic inflammation in both humans and animal models *in vivo*, and changes in its expression have been associated with different disease outcomes in various inflammatory disease models (Lith and de Vries, 2021). Some studies have reported that Nur77 can exert inhibitory effects on inflammation *via* transcriptional regulation of NF-κB signaling in the nucleus (Li et al., 2015). However, we found that Nur77 exerts pro-inflammatory and pro-apoptotic effects through ER translocation and subsequently activation of ER stress after exposure to CSE. These results suggest that the subcellular localization of Nur77 is essential for its function, that increased localization of Nur77 in ER may be a reflection of its increased localization out of the nucleus, where it might indeed play an inhibitory role in NF-KB transcription, which is align well with its purported role. Previous studies of Nur77 translocation also demonstrated that Nur77 can induce apoptosis of tumor cells, cardiomyocytes, and other cells via binding to Bcl-2 on mitochondria (Lin et al., 2004; Liu et al., 2008). However, its role in inflammation and apoptosis of airway epithelial cell during COPD development has not been documented. Airway epithelial cells represent the first barrier against environmental damage, such as that caused by inhaling cigarette smoke, and inflammatory and apoptotic processes in these cells play a critical role in the pathogenesis of COPD (Roscioli et al., 2018). Thus, identifying the relevant signaling pathways and potentially druggable targets involved in these pathological processes is an essential step in the effective treatment of COPD-related airway inflammation and injury.

Findings in this study support that ER stress is activated by treatment with CSE *in vitro*, which in turn promotes cell inflammation and apoptosis in bronchial epithelial cells. Moreover, the ER stress activation process involves Nur77 translocation to the ER, which is reduced, along with ER stress, following treatment with B6. B6, a flavonoid derivative, has been shown to play a therapeutic role in ALI disease by binding with Nur77-LBD *in vivo* and *in vitro*. Our study confirms that B6, which exhibits low toxicity to airway epithelial cells, has an inhibitory effect on inflammation and injury of airway epithelial cells after CSE stimulation, suggesting the promise of its clinical application in airway diseases.

These results provide mechanistic insight into the role of Nur77 in CSE-related inflammation and apoptosis, and the possible application of B6 in the treatment of COPD. However, there are some shortcomings of our research that should be addressed. First, as a respiratory disease, it would be better to explore the efficacy of candidate drugs in animal models of COPD, which is focus of our ongoing and future research. Second, we have not investigated the regulatory and transport mechanisms responsible for determining the subcellular localization of Nur77, which are both necessary to fully understand the role of B6 in modulating Nur77 nuclear and ER localization processes. To address this issue, future studies will experimentally investigate the details of Nur77-B6 interactions.

## Conclusion

This study provides robust evidence supporting the role of Nur77 in COPD-related airway inflammation and apoptosis. Our findings demonstrate that increased expression and translocation of Nur77 to the ER leads to ER stress, inflammatory response, and activation of apoptosis in human bronchial epithelial cells exposed to cigarette smoke extract *in vitro*. Moreover, we identified B6, a flavonoid derivative, as a modulator of Nur77 accumulation and ER translocation in CSE-treated airway epithelial cells. These results underscore the potential of B6 as a therapeutic candidate for airway inflammation and injury, and suggest that it may represent an effective therapeutic option for alleviating the severity of COPD. Further studies are warranted to fully elucidate the therapeutic potential of B6 in COPD management.

## Data Availability

The original contributions presented in the study are included in the article/Supplementary Material, further inquiries can be directed to the corresponding authors.

## Supplementary material

Supplementary Figure S1 The time-dependent effect of B6 in 16-HBE cells. **(A–C)** Western Blot was used to detect the protein levels of Nur77 [**(A)**, quantified in **(B)**] and CHOP [**(A)**, quantified in **(C)**] after the treatment of B6 combined with 2% CSE at different time points. **(D–F)** Detecting the protein levels of BIP [**(D)**, quantified in **(E)**] and ATF4 [**(D)**, quantified in **(F)**] after the treatment of B6 combined with 2% CSE at different time points by Western Blot. **(G–H)** CCK8 was used to determine the viability of 16-HBE cells stimulated by 5% CSE with B6 at 6 h **(G)** or 12 h **(H)**. Data are least squares means ± standard errors. **p* < 0.05; ***p* < 0.01,****p* < 0.001.

Supplementary Figure S2 Effects of B6 on inflammation and apoptosis in BEAS-2B cells stimulated by CSE.**(A)** rt-PCR was used to detect the mRNA levels of *Nur77* after 2% CSE stimulation with different concentrations of B6 in BEAS-2B cells. **(B–E)** The mRNA levels of *IL-6* **(B)**, *IL-8* **(C)**, *IL-1b* **(D)** and *TNFa* **(E)** in BEAS-2B cells stimulated by CSE with different concentrations of B6. **(F)** CCK8 was used to determine the viability of BEAS-2B cells stimulated by 5% CSE with different concentrations of B6. Data are least squares means ± standard errors. **p* < 0.05; ***p* < 0.01,****p* < 0.001.
