# Supplementary figures and images for: Inhibition of Nur77 expression and translocation by compound B6 reduces ER stress and alleviates cigarette smoke-induced inflammation and injury in bronchial epithelial cells
Source: Front Pharmacol. 2023 Jun 19;14:1200110. doi: 10.3389/fphar.2023.1200110 (PMC10315657; doi:10.3389/fphar.2023.1200110)

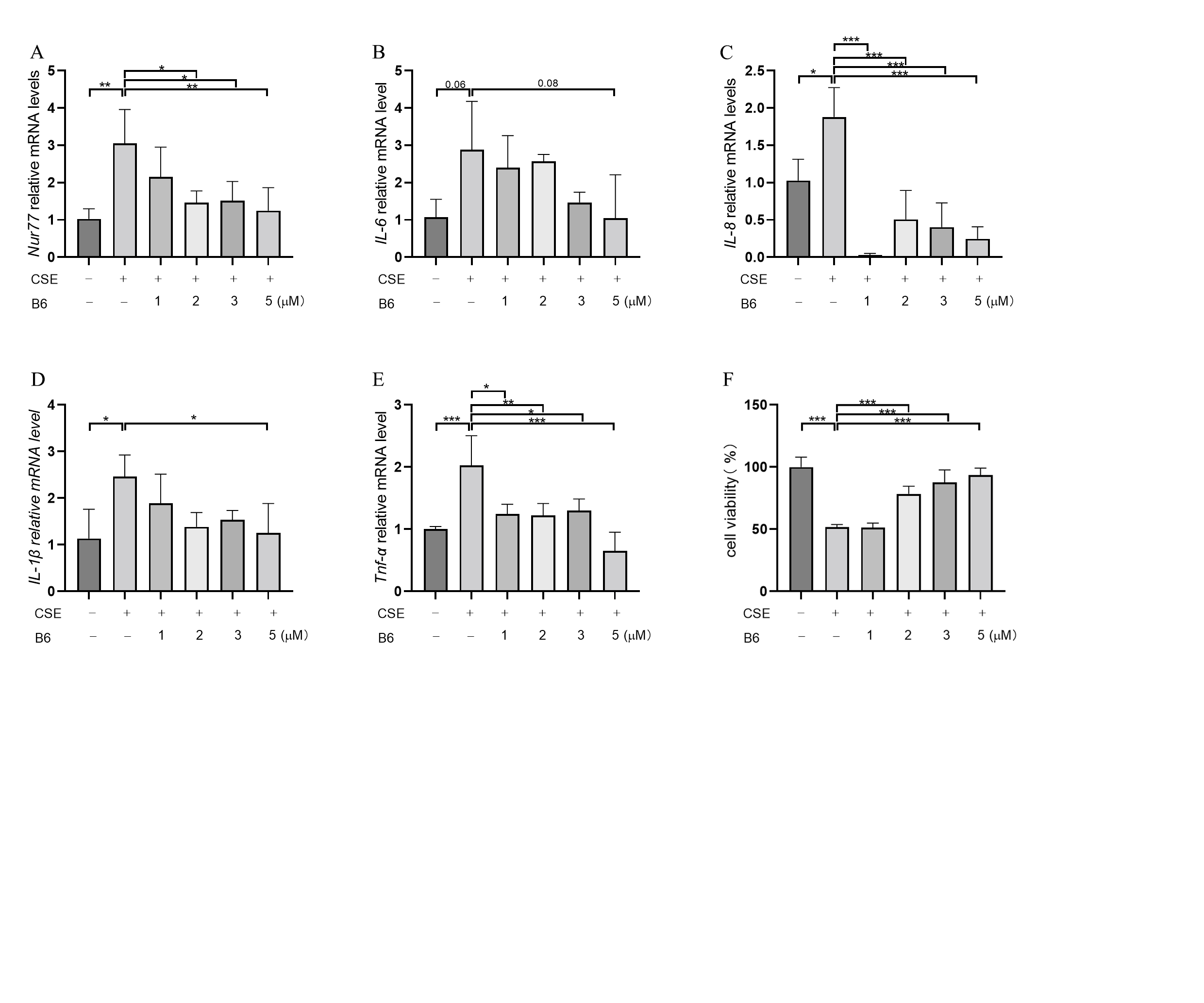

Supplement: Supplementary file 1 [file Image2.TIF]

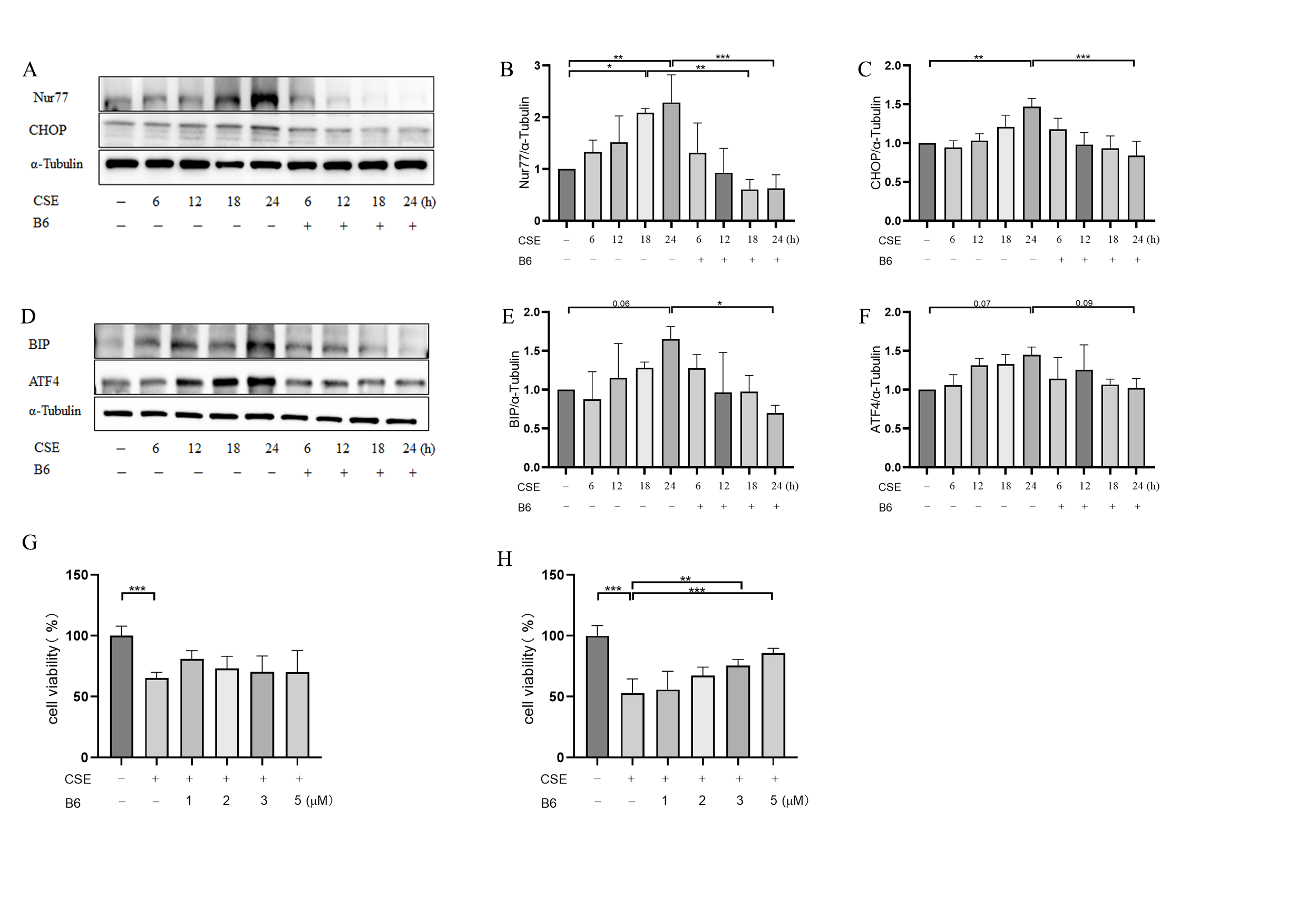

Supplement: Supplementary file 2 [file Image1.TIF]
